# Shape-Sensing Robotic-Assisted Bronchoscopic Microwave Ablation for Primary and Metastatic Pulmonary Nodules: Retrospective Case Series

**DOI:** 10.3390/diagnostics15243248

**Published:** 2025-12-18

**Authors:** Liqin Xu, Russell Miller, Mitchell Zhao, Grace Lin, Wenduo Gu, Niral Patel, Keriann Van Nostrand, Jorge A. Munoz Pineda, Bryce Duchman, Brian Tran, George Cheng

**Affiliations:** 1 Department of Interventional Pulmonology, Bronchoscopy, and Pleural Diseases, Division of Pulmonary, Critical Care & Sleep Medicine, UC San Diego Health, San Diego, CA 92103, USA; liqinxu2025@gmail.com (L.X.); r1miller@health.ucsd.edu (R.M.); wenduogu@stanford.edu (W.G.); nmp004@health.ucsd.edu (N.P.); kvannostrand@health.ucsd.edu (K.V.N.); jmunozpineda@health.ucsd.edu (J.A.M.P.); brduchman@health.ucsd.edu (B.D.); b6tran@health.ucsd.edu (B.T.); 2 Department of Pulmonary and Critical Care Medicine, Affiliated Hospital of Nantong University, Nantong 226001, China; 3 Department of Pulmonary Medicine, Naval Medical Center San Diego, San Diego, CA 92134, USA; 4 Department of Pathology, University of California, San Diego, CA 92093, USA; m3zhao@health.ucsd.edu (M.Z.); g4lin@health.ucsd.edu (G.L.)

**Keywords:** robotic-assisted bronchoscopy, microwave ablation, feasibility, safety, early outcomes

## Abstract

**Background:** Bronchoscopic thermal ablation has emerged as a minimally invasive therapeutic option for managing pulmonary nodules in patients unsuitable for surgery or radiotherapy. Robotic-assisted bronchoscopy (RAB) offers enhanced stability and precise navigation, potentially improving the safety and accuracy of bronchoscopic ablation. However, clinical data on RAB-guided microwave ablation (MWA) remains limited. Therefore, further evidence is needed to evaluate its feasibility, safety, and early therapeutic performance. **Methods:** We conducted a single-center retrospective feasibility study of shape-sensing RAB-guided MWA (ssRAB-MWA) for pulmonary nodules between October 2024 and September 2025. Eligible lesions (≤3.0 cm) included both primary lung cancers and metastatic nodules. All procedures were performed under general anesthesia using the ssRAB system integrated with cone-beam CT for intra-procedural confirmation. Technical success, safety outcomes, and short-term efficacy were assessed. **Results:** Nine patients (with 11 lesions: 3 primary, 8 metastatic) underwent ssRAB-MWA with 100% technical success. The median ablation time per nodule was 10 min (range, 1–26). One patient developed post-ablation pneumonia requiring hospitalization; no pneumothorax, major bleeding, or airway injury occurred. All lesions exhibited a transient increase in size immediately following MWA, followed by gradual reduction or stabilization over time. PET-CT evaluation demonstrated metabolic remission in primary lesions, with one patient achieving pathologic complete response after surgery. **Conclusions:** ssRAB-MWA appears to be a feasible and safe navigation-guided technique for small pulmonary lesions, offering encouraging early local control in both primary and metastatic lung cancers. This platform may expand the therapeutic spectrum of interventional pulmonology, bridging diagnosis and local therapy. Larger multicenter studies are warranted to validate long-term outcomes.

## 1. Introduction

Lung cancer remains the leading cause of cancer-related mortality worldwide [1], comprising both primary lung malignancies and pulmonary metastases from extrapulmonary neoplasms [2,3,4]. Early surgical resection offers the best chance for cure in primary lung cancer [5], yet many patients are deemed inoperable due to comorbidities, compromised pulmonary reserve, or multifocal disease [6]. In these cases, stereotactic body radiotherapy (SBRT) and percutaneous lung ablation have emerged as potentially curative local therapies [7,8]. Meanwhile, for pulmonary metastases, particularly in oligometastatic settings, local ablative therapies have likewise become an important adjunct to systemic therapy in improving local control and potentially prolonging survival [9].

Tumor ablation refers to the direct application of physical or chemical energy to a focal tumor to achieve substantial or complete destruction of tumor tissue. Thermal ablation techniques—such as radiofrequency ablation (RFA), microwave ablation (MWA), laser ablation (LA), and high-intensity focused ultrasound (HIFU)—induce cytotoxic temperatures within the target lesion to cause coagulative necrosis while sparing surrounding healthy parenchyma [10]. Among these, RFA and MWA are the most established modalities in clinical practice, each differing in energy source, heating mechanism, and tissue interactions. These minimally invasive techniques have demonstrated favorable safety profiles and allow repeated or combination treatments without precluding subsequent surgical resection due to lung sparing nature of thermal ablation [10]. Unlike RFA, which relies on electrical conduction, MWA generates heat through electromagnetic radiation at 915 MHz or 2.45 GHz, causing rapid molecular oscillation and uniform tissue heating that leads to coagulative necrosis [10,11,12]. Compared with RFA, MWA achieves higher intratumoral temperatures, produces larger and more uniform ablation zones, and is less affected by tissue impedance, carbonization, or the heat-sink effect near major vessels or aerated lung parenchyma [7,13]. These properties enable more consistent and predictable thermal profiles, making MWA particularly suitable for tumors in highly vascularized or aerated organs, such as the liver and lung [14,15,16].

Numerous studies have reported favorable local-control rates following MWA for pulmonary lesions [17,18,19]; however, percutaneous access for microwave ablation of lung lesions remains associated with relatively high rates of pneumothorax (often 30–60%) and occasional bleeding complications, particularly in patients with underlying emphysema or pleural involvement [17,20,21]. Recent investigations have explored transbronchial approaches, for example, electromagnetic-navigation bronchoscopy (ENB)-guided MWA has demonstrated early feasibility and safety in selected patients [22,23,24]. Recent innovations in bronchoscopic intervention for lung nodules have integrated RAB with intraprocedural cone-beam computed tomography (CBCT), enabling real-time confirmation of catheter or probe positioning and compensating for CT-to-body divergence [25,26,27]. Early RAB work—particularly when combined with CBCT—has shown in preclinical models improved targeting accuracy and reliable real-time probe confirmation [28], supporting the technical rationale for RAB-guided MWA while clinical outcome data are still emerging. However, comprehensive clinical evidence remains limited. A recent multi-center, prospective, single-arm study has reported a protocol evaluating the safety and feasibility of bronchoscopic MWA for peripheral lung cancer, and this trial is currently ongoing [29]. Additional validation in human studies is therefore warranted. Importantly, bronchoscopic access also offers potential advantages over transthoracic approaches, including the ability to perform invasive mediastinal staging in the same session and reduced risk of pleural complications.

In this context, our retrospective case series evaluated the feasibility, safety, and early outcomes of ssRAB-guided MWA for pulmonary nodules, thereby providing clinical evidence and new insights into the therapeutic potential of this approach. Collectively, these advances suggest that ssRAB-CBCT-guided ablation may represent a next-generation therapeutic modality, offering improved access to challenging anatomy, minimized procedural risk, and the potential for single-session diagnosis and therapy.

## 2. Materials and Methods

### 2.1. Study Design and Patients

This was a retrospective case series conducted at the University of California, San Diego Health from October 2024 to September 2025. Eligible patients included those with one or more peripheral pulmonary nodules (maximum diameter ≤ 3.0 cm), either primary lung cancer or metastatic lesions. Exclusion criteria included pulmonary nodules >3 cm, inability to tolerate bronchoscopy, significant uncorrected coagulopathy, or uncontrolled active infection. Patients undergoing ssRAB-guided diagnostic evaluation for pulmonary nodules were offered the option of same-session transbronchial microwave ablation (MWA). No additional procedural access or anesthesia beyond the diagnostic bronchoscopy was required. Multidisciplinary discussion for each patient was completed with respective oncology teams prior to the procedure. In patients with primary lung cancer, microwave ablation was performed concurrently with diagnostic biopsy according to patient preference, whereas in metastatic cases, microwave ablation was offered as a minimally invasive alternative to surgery. Patients were informed of the investigational nature of transbronchial MWA, including potential risks, benefits, and uncertainties compared with established treatments such as SBRT or percutaneous ablation. Those who elected to proceed were provided informed consent that included both diagnostic biopsy and transbronchial MWA. The decision to perform MWA was patient-driven preference after thorough informed discussion. All participants had adequate pulmonary reserve and no contraindications to bronchoscopy or ablation.

The study was conducted according to the Declaration of Helsinki (revised in 2013). Data collection and analysis were approved by the UC San Diego Institutional Review Board (approval number: #812977, approval date: 11 July 2025).

### 2.2. Pre-Procedure Planning and Procedure

Procedures were performed using the Ion™ endoluminal robotic bronchoscopy system (Intuitive Surgical, Sunnyvale, CA, USA) integrated with cone-beam CT (Siemens, Malvern, PA, USA) for intra-procedural imaging. High-resolution chest CT scans (≤1 mm slice thickness) were reconstructed for 3D airway mapping, and virtual pathways were planned using the Ion planning software (Intuitive Surgical, Sunnyvale, CA, USA).

Under general anesthesia and endotracheal intubation, the robotic catheter was navigated to the target nodule using CT-to-body registration and shape-sensing guidance. Radial EBUS and CBCT verification were performed to confirm tool-in-lesion. Transbronchial biopsy was obtained first, and rapid on-site cytologic evaluation (ROSE) confirmed malignancy before proceeding to ablation. Systematic EBUS staging was completed for the patient with primary lung cancer to ensure no nodal metastases. For patients who proceeded to resection, preoperative assessment followed standard institutional protocols, including updated cross-sectional imaging and routine cardiopulmonary evaluation, with operability determined through multidisciplinary review.

MWA was then performed using the MedWaves system (MedWaves Inc., San Diego, CA, USA). The ablation catheter was advanced through the working channel into the confirmed lesion. Temperature-controlled ablation was applied, with the target temperature typically set between 80 °C and 100 °C, and the duration adjusted according to lesion size and proximity to adjacent structures. A post-ablation CBCT scan was obtained 10 min after the ablation to verify the ablation zone and evaluate for immediate complications such as hemorrhage or pneumothorax. All procedures were performed by an experienced interventional pulmonology team.

A treatment “session” was defined as a single bronchoscopic procedure performed under one anesthetic event. Only one target was treated per session. Multiple cycles applied to the same lesion within a session did not represent separate sessions and did not constitute bracketing or treatment of multiple distinct lesions.

### 2.3. Post-Procedure Care and Follow-Up

Patients were monitored in the recovery unit, and chest imaging (chest X-ray or CT) was obtained within 24 h to detect immediate complications such as pneumothorax or bleeding. Clinical and radiologic follow-up was scheduled at 1, 3, 6, and 12 months, including contrast-enhanced CT to evaluate nodule reduction, residual enhancement, recurrence, and adverse events such as bronchial injury, atelectasis, or pneumonia. Actual follow-up intervals could vary based on patients’ clinical status, oncologic treatment schedules, and provider or patient preference.

The primary endpoints were technical success and safety. Technical success was defined as the successful placement of the microwave catheter within the target lesion and complete delivery of the planned ablation energy, confirmed by qualitative CBCT assessment. Safety was evaluated by monitoring and documenting all treatment-related adverse events occurring within 30 days after the procedure, which were graded according to the Common Terminology Criteria for Adverse Events (CTCAE, version 4.0).

Secondary endpoints included the evaluation of MWA impact on subsequent surgery for operable lesions, as well as exploratory efficacy measures such as early local tumor response, radiographic changes on follow-up imaging, and 3-month progression-free survival (PFS) rate. For surgically resected lesions, pathologic complete response (pCR) was defined as the absence of viable tumor cells in the ablation zone on postoperative histopathology. Metabolic complete response (mCR) was defined as the absence or marked reduction in FDG uptake on post-ablation PET-CT imaging. For inoperable or metastatic lesions, the local control rate at 3 months was assessed by chest CT, defined as the absence of lesion enlargement or new enhancement.

### 2.4. Statistical Analysis

Descriptive statistics were used (mean ± standard deviation or median [range] for continuous variables; frequency/percentage for categorical variables). The Wilcoxon matched-pairs signed rank test was used to compare pre- and post-ablation lesion sizes. A *p*-value < 0.05 was considered statistically significant.

## 3. Results

### 3.1. Patient and Lesion Characteristics

**Figure 1:**
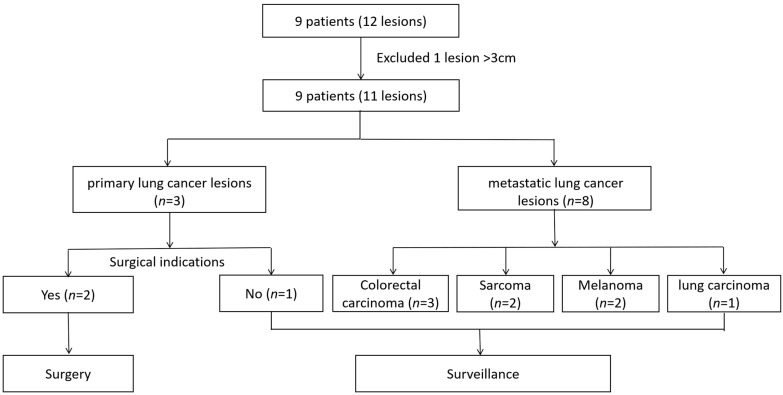
Patients who underwent microwave ablation, histopathology, and clinical management pathway.

A total of nine patients (11 lesions) underwent shaping sensing RAB-guided microwave ablation. Baseline patient and lesion characteristics are summarized in Table 1. The mean patient age was 68 ± 11.0 years (range, 50–79 years), and 33.3% were male. Among the treated lesions, 27.3% (3/11) were primary lung cancers, and 72.7% (8/11) were metastatic lesions, including three from colorectal carcinoma, one dedifferentiated liposarcoma, one osteosarcoma, two melanomas, and one metastatic lung carcinoma. The mean lesion diameter was 12.1 ± 5.7 mm (range, 7.0–24.0 mm). Lesions were located in the upper lobe (54.5%), middle lobe (27.3%), and lower lobe (18.2%), with detailed segmental distribution summarized in Table 1. All tumors underwent transbronchial needle biopsy or cryobiopsy prior to ablation, and ROSE confirmed malignancy before proceeding with MWA during the same anesthesia session. The overall study flow is illustrated in Figure 1.

### 3.2. Technical Outcomes

Of the nine patients, two had two pulmonary lesions each, and the remaining seven had one lesion treated with microwave ablation. Technical success was achieved in all 11 lesions (100%), as shown in Supplementary Table S1. A total of 14 ablation cycles were performed. Two patients required more than one cycle within the same bronchoscopic session to ensure adequate coverage—two cycles in one patient and three cycles in another. All cycles were successfully completed according to the planned ablation protocol. The median ablation time per lesion was 10 min (range, 1–26 min), with a mean duration of 10.4 ± 7.1 min.

### 3.3. Safety and Complications

Procedure-related complications associated with microwave ablation are presented in Table 2. The most frequently observed adverse event was chest/pleuritic pain, occurring in 33.3% of patients (3/9), all of which were CTCAE Grade 1. Throat pain, fatigue, and cough were also reported; these were mild (Grade 1), transient, and resolved with observation alone. One patient (Case 4) developed post-ablation pneumonia requiring short-term hospitalization and intravenous antibiotics (CTCAE Grade 3). One additional case of pneumonia occurred but was not clearly attributable to the MWA procedure. Among patients who subsequently underwent surgery, one developed a postoperative pleural effusion, and cytology was negative for malignant cells. No pneumothorax, airway injury, clinically significant hemoptysis/bleeding ≥ Grade 3, or treatment-related mortality occurred.

All adverse events were Grade 1–2. No serious adverse events (SAEs) or Grade ≥ 4 toxicities were observed.

### 3.4. Imaging Evolution After Ablation

**Figure 2:**
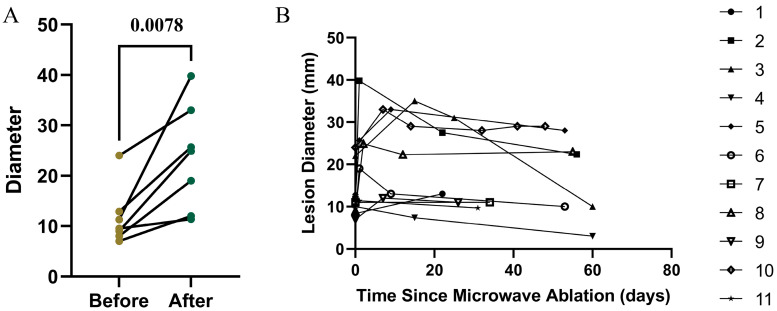
Temporal change in lesion size during the first two months after microwave ablation. (**A**) Change in lesion size within the first 7 days after MWA. (**B**) Change in size of all 11 lesions during the first two months after MWA.

Seven pulmonary lesions underwent follow-up chest CT within 7 days after MWA, as shown in Figure 2A, the treated lesions showed an increase size in the short-term apparent size compared with pre-ablation baseline (*p* = 0.0078), likely reflecting acute inflammatory response and peri-lesional edema. With continued follow-up, the ablation zones progressively decreased in size. Figure 2B shows the temporal size evolution of all 11 lesions within the first two months after MWA, highlighting this typical pattern of initial enlargement followed by gradual regression.

**Figure 3:**
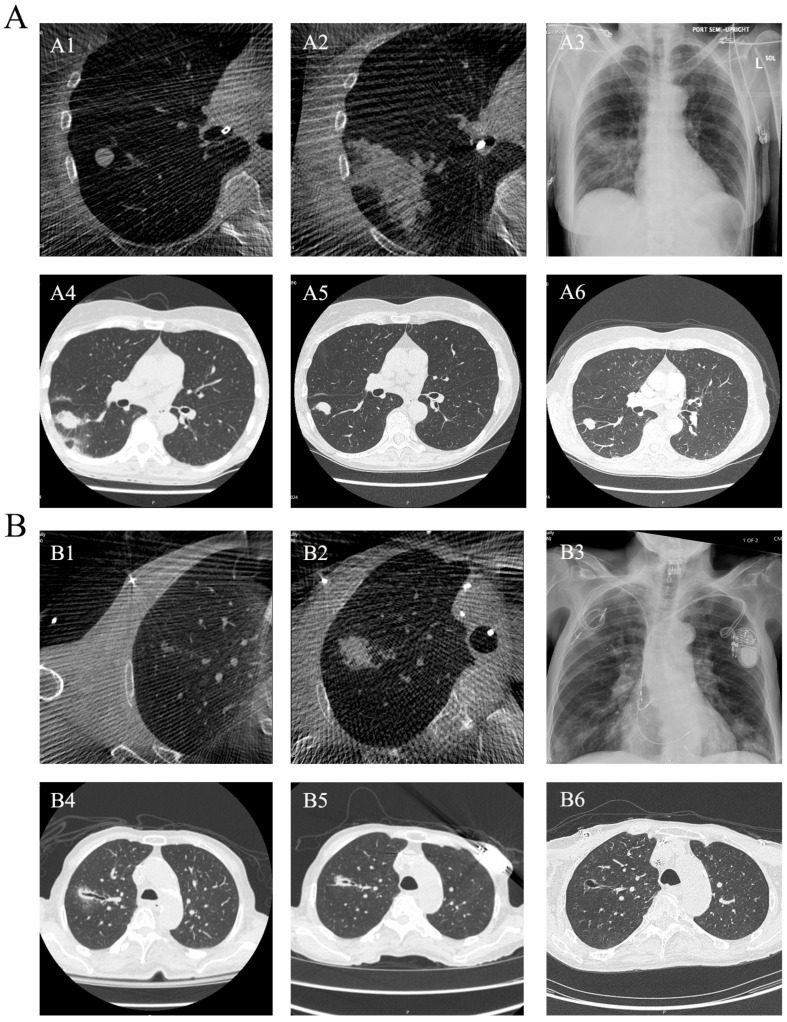
Representative temporal imaging evolution following ssRAB-MWA during the first two months. (**A**) Serial CT/CBCT images from Case 2 demonstrating lesion appearance before MWA (**A1**), immediately after MWA (**A2**), 1 h post-MWA (**A3**), 1 day post-MWA (**A4**), 22 days post-MWA (**A5**), and 56 days post-MWA (**A6**). (**B**) Serial CT/CBCT images from Case 8 demonstrating lesion appearance before MWA (**B1**), immediately after MWA (**B2**), 1 h post-MWA (**B3**), 2 days post-MWA (**B4**), 12 days post-MWA (**B5**), and 55 days post-MWA (**B6**).

Figure 3 illustrates representative temporal imaging evolution following robotic-assisted bronchoscopy-guided microwave ablation. Sequential CT/CBCT obtained before ablation, immediately post-ablation, and during serial follow-up within 2 months demonstrates characteristic morphological changes in the ablation zone. Immediately after ablation, CBCT typically showed a well-defined ground-glass opacity surrounding the original nodule, consistent with acute thermal injury, tissue edema, and early inflammatory change. At 1–2 days, perilesional edema partially regressed. By 2–3 weeks, peripheral inflammatory reaction progressively subsided, and the ablation zone became smaller and more fibrotic in appearance. During continued follow-up, the ablated lesion gradually shrinkage over time; in some cases, central cavitation developed, which has been described after thermal ablation and may reflect complete necrosis with airway drainage of liquefied tissue.

In Figure 3A, the lesion underwent three sequential MWA sessions—the immediate post-procedural CBCT demonstrated extensive acute inflammatory change, which gradually contracted and became smaller over time. In Figure 3B, the ablation bed evolved into a central cavity within 2 months. This temporal evolution pattern was consistently observed in lesions with technically successful ablation and no radiographic evidence of local recurrence during early follow-up, consistent with post-ablative healing patterns described in the percutaneous thermal ablation literature.

### 3.5. Efficacy of Microwave Ablation for Primary Lung Cancer

Among the 11 treated lesions, three were primary lung cancers, all of which achieved technical success. Two patients (Case 5 and Case 7) subsequently underwent surgical resection of the ablated lesions, while Case 6, who was not a surgical candidate due to cardiac dysfunction, was managed with imaging follow-up only based on multidisciplinary team (MDT) discussion and has remained radiographically stable during the observation period. All three patients underwent post-ablation PET-CT evaluation. Two lesions (Case 6 and Case 7) showed no FDG uptake, and one lesion (Case 5) demonstrated mild residual uptake. The temporal imaging evolution of these three primary lung cancer lesions is presented in Supplementary Figure S1.

**Figure 4:**
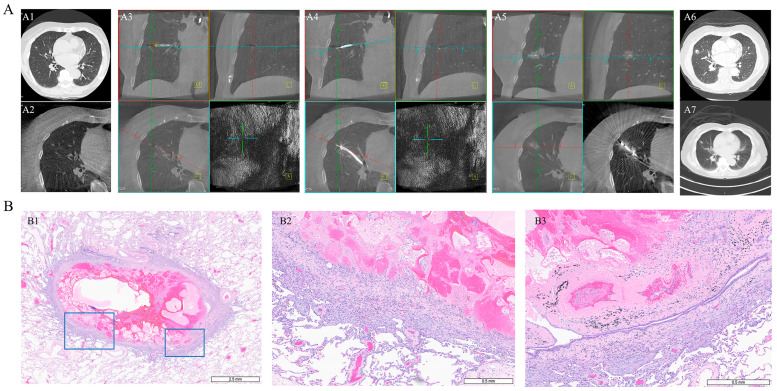
Serial chest CT imaging before/after MWA and corresponding post-surgical pathology. (**A**) Serial CT/CBCT images from Case 7 demonstrating lesion evolution surrounding microwave ablation. (**A1**) Chest CT before MWA; (**A2**,**A3**) CBCT confirming lesion location before MWA; (**A4**) biopsy prior to ablation; (**A5**) confirmation of MWA catheter position; (**A6**) CT at 7 days post-MWA; (**A7**) CT at 26 days post-MWA. (**B**) Histopathology of the resected ablation zone after surgery, demonstrating ablation-induced changes. (**B1**) ×8 magnification; (**B2**) ×40 magnification; (**B3**) ×50 magnification.

Interestingly, Case 7 demonstrated a pathologic complete response (pCR), with no viable tumor cells detected in the resected specimen, as shown in Figure 4, whereas Case 5 showed less than 5% residual viable tumor cells within the ablation zone.

### 3.6. Early Efficacy of Microwave Ablation for Metastatic Lung Lesions

**Figure 5:**
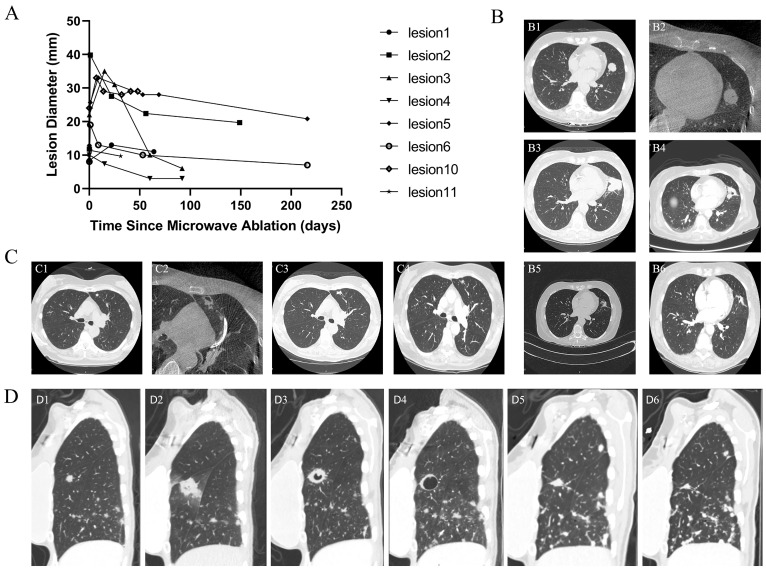
Early radiologic evolution of metastatic pulmonary nodules following ssRAB-guided microwave ablation. (**A**) Size-change curves of eight metastatic pulmonary nodules following MWA. (**B**) Serial chest CT images from Case 3- LB5 lesion. (**B1**) Pre-MWA baseline; (**B2**) Immediately post-MWA; (**B3**) 15 days post-MWA; (**B4**) 25 days post-MWA; (**B5**) 60 days post-MWA; (**B6**) 110 days post-MWA. (**C**) Serial chest CT images from Case 3- LB3 lesion. (**C1**) Pre-MWA baseline; (**C2**) Immediately post-MWA; (**C3**) 15 days post-MWA; (**C4**) 110 days post-MWA. (**D**) Serial chest CT images from Case 4. (**D1**) Pre-MWA baseline; (**D2**) 1 day post-MWA; (**D3**) 53 days post-MWA; (**D4**) 125 days post-MWA; (**D5**) 216 days post-MWA; (**D6**) 283 days post-MWA.

Among the eight patients, all continued to receive treatment for their primary malignancies during the follow-up period. The imaging evolution of the eight metastatic lesions is demonstrated in Figure 5A, where all lesions exhibited a transient post-MWA enlargement, followed by gradual reduction or stabilization over time. At 3 months, all evaluable treated lesions demonstrated local control, corresponding to a 100% lesion-based local control rate.

Six ablated lesions developed central cavitation on follow-up CT, a typical post-ablation change reflecting tissue necrosis and drainage into adjacent airways. With continued follow-up, the cavitary changes gradually decreased and resolved, accompanied by progressive lesion shrinkage. Figure 5B,C demonstrates the longitudinal evolution of case 3 (2 lesions in the left upper lobe) over the follow-up period. Two patients experienced systemic disease progression related to their primary malignancy, with new or enlarging extrathoracic metastases; however, the ablated pulmonary lesions remained radiographically stable without local recurrence or regrowth (Figure 5D).

## 4. Discussion

Thermal ablation for pulmonary nodules has emerged as an alternative treatment option for patients who are not candidates for surgery or radiotherapy. Among the available techniques, percutaneous microwave ablation has been widely applied and shown favorable local control rates in previous studies; however, it is also associated with a relatively high incidence of complications, such as pneumothorax and bleeding [30,31,32].

In recent years, investigators have begun to explore the feasibility and safety of bronchoscopic approaches for delivering ablative energy to lung nodules. Over the past decade, the concept of bronchoscopic ablation has evolved from early experimental attempts using navigation bronchoscopy to deliver ablative probes, toward more advanced and integrated systems [33]. Early clinical applications of bronchoscopic MWA under navigation guidance have shown promise. Chan et al. reported a 30-case series of transbronchial MWA via ENB guidance, establishing initial safety and feasibility in human lung nodules [33,34]. More recently, large retrospective studies of ENB-guided MWA have emerged, Huang et al. reported experience in multiple pulmonary nodules (MPNs), combining ENB ablation with video-assisted thoracoscopic surgery (VATS) in many cases, demonstrating favorable short-term safety and shrinkage of ablation zones over months [35]. Use of CBCT in ENB-guided transbronchial MWA significantly improved technical and ablation success rates (97.0% vs. 91.5%) without raising complications [36]. Concomitant multi-nodular transbronchial MWA in one session has also been shown to be feasible and safe, with no increase in complication rates and shorter cumulative anesthesia times [37]. However, ENB-based systems still contend with challenges such as catheter instability, drift, CT-to-body divergence affect, and the need for repeated imaging correction [27]. Robotic-assisted bronchoscopy potentially mitigates these issues by providing stable robotic-driven control, shape-sensing feedback, and more consistent instrument guidance. Reviews indicate that combining robotic bronchoscopy with ablative tools is feasible in principle and emerging in practice [27,38,39,40]. Robotic-assisted bronchoscopy platforms provide enhanced maneuverability, steadiness, and precise instrument positioning, facilitating interventions deeper into the periphery with greater confidence [27,39,41,42].

In this exploratory, single-arm, retrospective feasibility series, we performed shape-sensing robotic-assisted bronchoscopy (ssRAB)–guided microwave ablation (MWA) for pulmonary lesions, including three cases of primary lung cancer and eight cases of metastatic lung disease. Technically, all lesions achieved successful catheter placement and completion of the planned ablation, which is consistent with findings from preclinical studies evaluating the feasibility of bronchoscopic or robotic-assisted microwave ablation [28]. The initial animal study combining RAB and microwave ablation using the Neuwave™ Flex MWA system demonstrated favorable safety, with no peri- or post-procedural adverse events observed in 17 swine treated under CBCT guidance [28]. Building on this preclinical foundation, our current series represents one of the earliest clinical experiences of RAB-guided MWA for pulmonary lesions and offers preliminary human data supporting its technical feasibility and short-term safety.

In our series, ssRAB assisted MWA demonstrated a favorable safety profile. Only one patient developed post-ablation pneumonia that required hospitalization and antibiotic therapy, which may have been related to local inflammatory response or thermal injury to adjacent airways. No cases of pneumothorax, significant bleeding, or airway perforation were observed, and all other post-procedural symptoms-including cough, sore throat, and headache-were mild and self-limited. Importantly, there were no treatment-related deaths or major adverse events. Published data on transbronchial MWA likewise report low rates of severe complications. In the NAVABLATE study [17], transbronchial MWA of malignant lung nodules ≤ 30 mm achieved no deaths or pneumothorax, with only a 13.3% rate of grade ≥ 3 complications—demonstrating an overall acceptable safety profile [23], notably lower than the pooled complication rates reported for percutaneous thermal ablation, in which pneumothorax occurs in approximately 34% (95% CI 25.9–43.1%) following RFA or MWA [43]. Complementing these findings, device safety assessments using the Neuwave™ Flex system in animal models have confirmed the tolerability of microwave energy delivery through bronchoscopic platforms, with no peri-procedural or post-procedural adverse events observed across 17 swine undergoing RAB-guided MWA under CBCT assistance [28]. The integration of CBCT or intra-operative imaging has proven crucial for ensuring accuracy and safety. A 2025 study demonstrated that adding CBCT to ENB-guided MWA significantly improves tool-in-lesion confirmation and ablation success without increasing complication rates [36]. In robotic-assisted workflows, the enhanced catheter stability and precise control provided by the robotic platform, in combination with real-time CBCT confirmation, allows more predictable lesion targeting and minimizes unintended thermal spread. These factors together suggest that RAB-guided MWA may offer a safer and more controlled approach for the management of pulmonary nodules, particularly in patients at higher risk for pneumothorax or bleeding complications.

Interestingly, in our primary lung cancer subgroup (*n* = 3), post-ablation PET-CT provided early metabolic readouts of local response. Two lesions showed no FDG uptake, one achieving pathologic complete response after surgery while the lesion with residual uptake demonstrated viable tumor on resection, consistent with reports that persistent hypermetabolism indicates residual disease [44,45,46,47]. These findings suggest that PET-CT may serve as a practical readout of early ablation efficacy and help guide subsequent surgical decisions. However, these observations are anecdotal within this small feasibility cohort and could not be interpreted as directly comparable to established surgical or SBRT outcomes. Additional data from larger studies will be needed to validate these preliminary findings.

Beyond direct thermal cytotoxicity, microwave ablation (MWA) may remodel the metabolic–immune axis within the tumor microenvironment. Several published studies suggest thermal injury and coagulative necrosis may lead to the release of tumor-associated antigens and damage-associated molecular patterns (DAMPs), including calreticulin exposure, extracellular ATP, high-mobility group box 1 (HMGB1), and heat-shock proteins (HSP70/90)—which collectively drives dendritic-cell activation and cytotoxic T-cell priming [48,49,50]. Preclinical studies have suggested that MWA-induced antigen release, may activate antigen-presenting cells and augment systemic antitumor immunity [51], while clinical investigations in lung cancer have hinted abscopal-like immune responses, suggesting that localized hyperthermic injury may trigger systemic immune activation beyond the ablation zone [52]. In parallel, metabolic alterations following ablation—such as reduced glucose avidity and glycolytic flux on FDG-PET—may reflect a decrease in lactate accumulation, thereby relieving lactate-mediated immunosuppression and enhancing effector T-cell function. Together, these effects suggest that MWA may induce both metabolic and immunologic remodeling, providing a potential rationale for future investigations of combining bronchoscopic ablation with immunotherapy or metabolic interventions.

In patients with metastatic pulmonary nodules, short-term follow-up observed durable local control within the ablation zone at 3 months post-procedure. During the early post-ablation period, transient inflammatory changes such as cavitation and peripheral consolidation were frequently observed, followed by gradual fibrosis and cavity resolution over time, consistent with expected healing dynamics after thermal ablation. These temporal changes were radiographically similar to those reported after percutaneous MWA, where cavitation and parenchymal contraction typically represent benign post-ablative remodeling rather than recurrence [30]. Importantly, even in cases where the primary or extrapulmonary disease progressed, the ablated pulmonary lesions in our cohort remained radiographically stable without evidence of local recurrence. This observation supports the role of bronchoscopic MWA as a means of achieving durable local tumor control for metastatic nodules, potentially contributing to intrathoracic disease stability and delaying systemic progression. Nevertheless, longer-term follow-up and larger multicenter studies are warranted to determine the durability of local control and potential survival benefit in this population.

This study has several limitations. First, the study cohort was small and derived from a single institution, reflecting the exploratory nature of early robotic-assisted bronchoscopic MWA experience. Second, ablation parameters—including treatment settings, duration, and probe positioning—have not yet been standardized for transbronchial application, which may influence ablation volume and reproducibility across centers. Third, the relatively short follow-up precludes definitive conclusions regarding long-term durability, local recurrence, and survival outcomes. Additionally, although post-ablation PET-CT appeared useful for assessing early metabolic response, its accuracy in predicting complete ablation or guiding surgical decision-making requires validation in larger cohorts. Furthermore, there is a learning curve to navigation bronchoscopy, use of CBCT, and MWA that is inherent to any procedure. As such, individual proceduralist experience will often influence the efficacy of the MWA procedure, which will further limit the generalizability of our study. Finally, potential biological markers of complete ablation—such as serum inflammatory mediators, circulating tumor DNA (ctDNA), or metabolic–immune signatures—remain to be elucidated and may provide mechanistic insight into treatment efficacy. Future multicenter, prospective studies with standardized imaging and biomarker integration are warranted to validate these findings and establish optimized procedural protocols.

## 5. Conclusions

Shape-sensing robotic-assisted bronchoscopic microwave ablation (ssRAB-MWA) enables a minimally invasive, navigation-guided treatment approach for small pulmonary lesions-both primary and metastatic, especially when guided by CBCT. The present findings suggest that ssRAB-MWA is technically feasible and appears safe, with encouraging short-term local control, particularly in patients unsuitable for surgery or radiotherapy.

For primary lung cancer, ssRAB-MWA may represent a potential local therapeutic option or bridge strategy to surgery, while for metastatic disease, it may serve as an adjunct to systemic therapy to maintain intrathoracic stability. Integration of intra-procedural imaging such as cone-beam CT enables confirmation of accurate probe placement, enhancing procedural precision. Future multicenter prospective studies—including the recently published protocol evaluating bronchoscopic MWA for peripheral lung cancer [29]—will be essential to generate outcome data. Standardized ablation parameters, extended follow-up, and incorporation of metabolic and immunologic biomarkers will further clarify long-term efficacy and help refine patient selection.

## Figures and Tables

**Table 1:** Clinical characteristics of patients and lung nodules (*N* = 9) (11 lesions).

Characteristic	Value
**No. of patients**	9
**No. of nodules**	11
**Patient characteristics**	
**Age (year)**	
Mean	68.0 ± 11.0
Range	50–79
**Sex (patients)**	
Male	3 (33.3%)
Female	6 (66.7%)
**Smoke (patients)**	
Yes	3 (33.3%)
No	6 (66.7%)
**Lung nodules characteristics**	
**With biopsy**	11 (100%)
**Pre-ablation pathological diagnostic yield (%, *n*/*N*)**	11 (100%)
**Histology (nodules)**	
Primary lung cancer	3 (27.3%)
Metastatic cancer	8 (72.7%)
**Location**	
RUL	4 (36.3%)
RLL	2 (18.2%)
LUL	2 (18.2%)
RML	3(27.3%)
**Nodule type**	
Solid	7 (63.6%)
Mixed	4 (36.4%)
**Nodule volume (mm^3^)**	
Mean	1463 ± 1686
Range	67.0–5027.0
**Nodule size (maximum diameter mm)**	
Mean	12.1 ± 5.7
Range	7.0–24.0
**EBUS-GS**	
Concentric	4 (36.4%)
Eccentric	6 (54.5%)
Not visible	1 (9.1%)
**Ablation time (minute)**	
Mean	10.4 ± 7.1
Range	1.0–26.0

**Table 2:** Adverse events of microwave ablation within 1 month of the procedure.

AE Type, *n* (%)	Results (*n* = 9)
Throat pain	22.2% (2/9)
Peri-procedural pneumothorax	0% (0/9)
Peri-procedural pneumonia	11.1% (1/9)
Bloody sputum/hemoptysis	0% (0/9)
Shortness of breath	11.1% (1/9)
Chest pain/pleuritic pain	33.3% (3/9)
Tired	22.2% (2/9)
Cough	22.2% (2/9)

## Data Availability

The original contributions presented in this study are included in the article/Supplementary Materials. Further inquiries can be directed to the corresponding author.

## Supplementary Materials

The following supporting information can be downloaded at https://www.mdpi.com/article/10.3390/diagnostics15243248/s1, Figure S1: Size-change curves of primary lung cancer nodules following microwave ablation; Table S1: Microwave ablation details for 11 lung nodules in 9 patients.
