# Supplementary figures and images for: Shape-Sensing Robotic-Assisted Bronchoscopic Microwave Ablation for Primary and Metastatic Pulmonary Nodules: Retrospective Case Series
Source: Diagnostics (Basel). 2025 Dec 18;15(24):3248. doi: 10.3390/diagnostics15243248 (PMC12731843; doi:10.3390/diagnostics15243248)

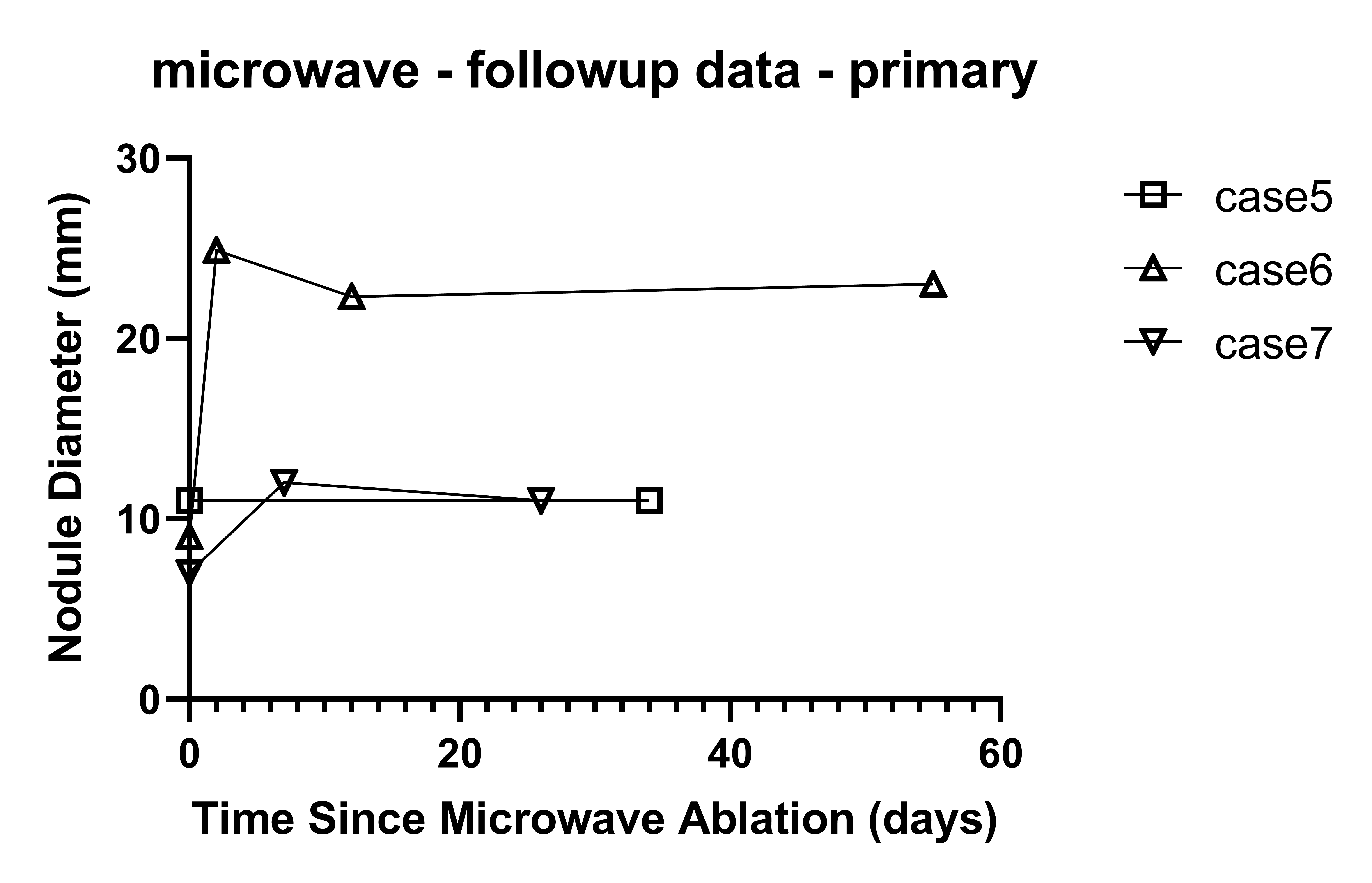

Supplement: Supplementary file 1 [file diagnostics-15-03248-s001.zip › Supplementary Figure S1.tif]
